# Comparison of the prognostic values of preoperative inflammation-based parameters in patients with breast cancer

**DOI:** 10.1371/journal.pone.0177137

**Published:** 2017-05-10

**Authors:** Hideya Takeuchi, Hirohumi Kawanaka, Seiichi Fukuyama, Nobuhide Kubo, Shoji Hiroshige, Tokujiro Yano

**Affiliations:** 1 Department of Surgery, National Hospital Organization Beppu Medical Center, Beppu, Oita, Japan; 2 Clinical Research Institute, National Hospital Organization Beppu Medical Center, Beppu, Oita, Japan; University of North Carolina at Chapel Hill School of Medicine, UNITED STATES

## Abstract

Peripheral blood-derived inflammation-based markers, including C-reactive protein (CRP), neutrophil-to-lymphocyte ratio (NLR), lymphocyte-to-monocyte ratio (LMR), and platelet-to-lymphocyte ratio (PLR) are indicators of prognosis in various malignant tumors. The present study aimed to identify the inflammation-based parameters that are most suitable for predicting outcomes in patients with breast cancer. Two hundred ninety-six patients who underwent surgery for localized breast cancer were reviewed retrospectively. The association between clinicopathological factors and inflammation-based parameters were investigated. Univariate and multivariate Cox regression analyses were performed to identify independent prognostic indicators associated with disease-free survival (DFS). The NLR level correlated significantly with tumor size (P<0.05). The PLR level correlated with the expression of estrogen receptor and lymph node involvement (P<0.05). Univariate analysis revealed that lower CRP and PLR values as well as tumor size, lymph node involvement, and nuclear grade were significantly associated with superior DFS (CRP: P<0.01; PLR, tumor size, lymph node involvement, and nuclear grade: P<0.05). On multivariate analysis, CRP (hazard ratio [HR]: 2.85, 95% confidence interval [CI]: 1.03–7.88, P<0.05), PLR (HR: 2.61, 95% CI: 1.07–6.36, P<0.05) and nuclear grade (HR: 3.066, 95% CI: 1.26–7.49, P<0.05) were significant prognostic indicators of DFS in patients with breast cancer. Neither LMR nor NLR significantly predicted DFS. Both preoperative CRP and PLR values were independently associated with poor prognosis in patients with breast carcinoma; these were superior to other inflammation-based scores in terms of prognostic ability.

## Introduction

Breast cancer is the most frequently diagnosed cancer in women worldwide. As a result of improved treatments and earlier detection, the mortality rate associated with breast cancer has decreased in most Western countries in recent years. However, breast cancer remains the third-leading cause of cancer-related death in Europe and the United States [1].

Identification of prognostic indicators, which allow for the proper risk stratification of cancer patients and the selection of appropriate treatment, remains the subject of intense investigation in breast cancer. Recently, molecular kits (e.g., Oncotype Dx and Mammaprint) have been used to predict prognostic information in patients with breast cancer; however, The National Health Insurance does not support the routine evaluation for breast cancer using these kits in Japan because of their high cost and limited regional availability [2]. Therefore, the evaluation of patients’ prognoses using simple, inexpensive, and easily interpretable clinical parameters is an unmet need.

It is well known that systemic inflammatory responses play an important role in cancer progression [3, 4]. The tumor microenvironment, which is regulated by inflammatory cells, is clearly involved in the neoplastic process, including the stimulation of proliferation, migration, and survival [3]. Systemic inflammatory responses are characteristically reflected by changes in the relative levels of C-reactive protein (CRP) and circulating white blood cells, resulting in a change in the proportions of neutrophils, lymphocytes, and monocytes. These factors have been investigated as surrogate markers in tumor biology [3]. Therefore, peripheral blood-derived inflammation-based parameters, such as the lymphocyte-to-monocyte ratio (LMR), neutrophil-to-lymphocyte ratio (NLR), and CRP levels (all of which can be assessed at lower costs and less complicated expenditures compared to complex molecular markers) have been reported to be independent prognostic biomarkers in various types of tumors [3, 5–7]. We have also shown that the platelet-to-lymphocyte ratio (PLR) is a significant prognostic marker in patients with breast cancer [8].

To the best of our knowledge, the optimal inflammation-based parameter for predicting the outcome of patients with breast cancer among the many that have been revealed so far has not been elucidated. The purpose of this study was to compare the prognostic value of these inflammation-based parameters in patients with breast cancer.

## Patients and methods

### Patients

The medical records of 459 breast cancer patients who underwent surgery at the National Hospital Organization Beppu Medical Center between April 2005 and December 2014 were reviewed and retrospectively analyzed. The exclusion criteria were: (i) patients with distant metastases at initial presentation (n = 10), carcinoma in situ (n = 50), bilateral breast carcinoma (n = 19), and male breast carcinoma (n = 5); (ii) patients with comorbidities that affected serum CRP levels, including infection (n = 1), collagen disease (n = 1), and liver cirrhosis (n = 7); and (iii) patients with incomplete laboratory data (n = 68). Ultimately, 296 patients were eligible for analysis and were reviewed retrospectively.

Adjuvant therapy was administered according to the St. Gallen recommendations [9]. Follow-up care was performed at regular intervals (3-month intervals during years 1–5 and, 6-month intervals during years 5–10 post-diagnosis). Follow-up investigations included clinical examinations, laboratory data analyses (including carcinoembryonic antigen and carbohydrate-antigen 15–3), and radiological assessment (computed tomography and mammography) every 6–12 months.

### Pathological characteristics

Pathological data were reviewed to determine the tumor size, nuclear grade, lymph node involvement, hormone receptor status, and human epidermal growth factor receptor 2 (HER2) status. Estrogen receptor and progesterone receptor statuses were evaluated via immunohistochemistry (IHC). Tumors with nuclear expression levels ≥1% were deemed positive. HER2 status was assessed via IHC or fluorescence in situ hybridization and was considered positive if the IHC score was 3 or if its expression was at least 2.2-fold stronger than the CEP-17 signal in tumor cells [10].

### Inflammatory parameters

Blood samples were obtained via peripheral venous puncture before the initiation of any treatment modality. Serum CRP levels were measured routinely with an automatic nephelometer (TBA-c16000; Toshiba Corporation, Tokyo, JAPAN); neutrophils, platelets, lymphocytes and monocytes were also measured routinely with a different automatic nephelometer (XE-5000; Sysmex Corporation, Tokyo, JAPAN) according to the manufacturer’s instructions. Assays were performed by medical technicians who were unaware of the study subjects’ clinical information. The NLR, LMR, and PLR were calculated as the absolute neutrophil count divided by the absolute lymphocyte count, absolute lymphocyte count divided by the absolute monocyte count, and absolute platelet count divided by absolute lymphocyte count, respectively.

### Statistical analysis

The primary endpoint of the study was disease-free survival (DFS), which was defined as the interval between the date of any initial treatment and the first detection of disease relapse.

Receiver operating characteristics (ROC) curve analysis was performed to determine the optimal cut-off values for prognostic factors related to DFS. The score with maximum sensitivity and specificity was selected as the approximate cut-off value for each parameter. The area under the curve was also calculated. Comparisons between groups were performed using Fisher’s exact test. The Kaplan-Meier method was used to calculate the DFS rates.

Univariate and multivariate analyses were performed for prognostic factors using the Cox proportional hazards model. Variables that proved to be significant on univariate analysis were subsequently subjected to a multivariate Cox proportional hazards model. Hazard ratios (HRs) estimated using the Cox analysis are reported as relative risks with corresponding 95% confidence intervals (CIs). All statistical analyses were performed using EZR (Saitama Medical Center, Jichi Medical University, Saitama, Japan), a graphical user interface for R (The R Foundation for statistical Computing, Vienna, Austria). EZR is a modified version of R Commander that is designed to include the statistical functions frequently used in biostatistics [11]. A P-value <0.05 was considered significant.

### Data collection

This study, including the opt-out consent method, was approved by the Institutional Ethical Committee of the National Hospital Organization, Beppu Medical Center (2016–13). All medical data from the participants were anonymized and compiled; it was therefore determined that there was no risk of individually participating patients being identified. The study plan and choice to freely refuse participation were announced through the hospital bulletin at the National Organization Beppu Medical Center. Patients were considered to have consented to the study if they did not request to refuse participation.

## Results

### Patient characteristics

The baseline characteristics of the patients are shown in Table 1.

**Table 1:** Basic characteristics of the enrolled patients.

Variables	No. (%)
Age (years)	
<50	61 (21)
≥50	235 (79)
Tumor size (mm)	
<20	143 (48)
≥20	153 (52)
Estrogen receptor	
Negative	43 (15)
Positive	253 (85)
Progesterone receptor	
Negative	74 (25)
Positive	222 (75)
HER2	
Negative	49 (17)
Positive	247 (83)
Lymph node involvement	
Negative	209 (71)
Positive	87 (29)
Nuclear grade	
1–2	203 (69)
3	93 (31)
CRP (mg/dL)	
<0.37	268 (91)
≥0.37	28 (9)
LMR	
<4.56	46 (16)
≥4.56	250 (84)
NLR	
<2.06	159 (54)
≥2.06	137 (46)
PLR	
<162.28	212 (72)
≥162.28	84 (28)

Abbreviations: No, number; HER2, human epidermal growth factor receptor 2; CRP, C-reactive protein; LMR, lymphocyte-to-monocyte ratio; NLR, neutrophil-to-lymphocyte ratio; PLR, platelet-to-lymphocyte ratio.

The optimal cut-off values that were determined by ROC for CRP, LMR, NLR, and PLR are shown in Table 2. Based on these cut-off values, we separated the patients into two groups (low-value vs. high-value) in each category. Twenty-eight (9%), 250 (84%), 137 (46%), and 84 (28%) patients had high CRP, LMR, NLR, and PLR, respectively.

**Table 2:** Receiver operating characteristics analyses of inflammation-base parameters in patients with breast cancer.

Variables	Cut-off value	AUC (95% CI)	Specificity	Sensitivity
CRP (mg/dL)	0.37	0.58 (0.46–0.71)	0.92	0.27
LMR	4.56	0.54 (0.41–0.67)	0.85	0.27
NLR	2.06	0.56 (0.43–0.69)	0.55	0.64
PLR	162.28	0.61 (0.49–0.73)	0.74	0.5

Abbreviations: AUC, area under the curve; CI, confidence interval; HER2: human epidermal growth factor receptor 2; CRP: C-reactive protein; LMR: lymphocyte to monocyte ratio; NLR: neutrophil to lymphocyte ratio; PLR: platelet to lymphocyte ratio.

The relationships between inflammation-based scores and clinicopathological factors are summarized in Table 3. The NLR correlated significantly with tumor size (P<0.05). The PLR correlated with estrogen receptor expression and lymph node involvement (P<0.05).

**Table 3:** Association between inflammation-based parameters and clinicopathological factors in patients with breast cancer.

Variables	CRP			LMR			NLR			PLR		
	L	H	P	L	H	P	L	H	P	L	H	P
Age (y)												
<50	59	2	0.08	8	53	0.69	31	30	0.67	39	22	0.15
≥50	209	26		38	197		128	107		173	62	
TS												
<20	134	9	0.08	21	122	0.75	86	57	0.04	107	36	0.25
≥20	134	5		25	128		73	80		105	48	
ER												
(–)	38	5	0.58	5	48	0.65	25	18	0.62	37	6	0.03
(+)	230	23		41	212		134	119		175	78	
PgR												
(–)	68	6	0.82	9	65	0.46	38	36	0.69	51	23	0.55
(+)	200	22		37	185		121	101		161	61	
HER2												
(–)	222	25	0.59	38	209	0.83	135	112	0.53	181	66	0.17
(+)	46	3		8	41		24	25		31	18	
LN												
(–)	191	18	0.51	32	177	0.86	120	89	0.06	158	51	0.02
(+)	77	10		14	73		39	48		54	33	
NG												
1–2	183	12	1	30	172	0.61	111	91	0.45	143	59	0.68
3	83	9		16	76		46	46		68	24	

Abbreviations: CRP, C-reactive protein; LMR, lymphocyte-to-monocyte ratio; NLR, neutrophil-to-lymphocyte ratio; PLR, platelet-to-lymphocyte ratio; TS, tumor size; L, low inflammation-based parameter group; H, high inflammation-based parameter group; P, P-value; ER, estrogen receptor; PgR, progesterone receptor; HER2, human epidermal growth factor receptor 2; LN, lymph node involvement; NG, nuclear grade.

### Survival

**Fig 1:**
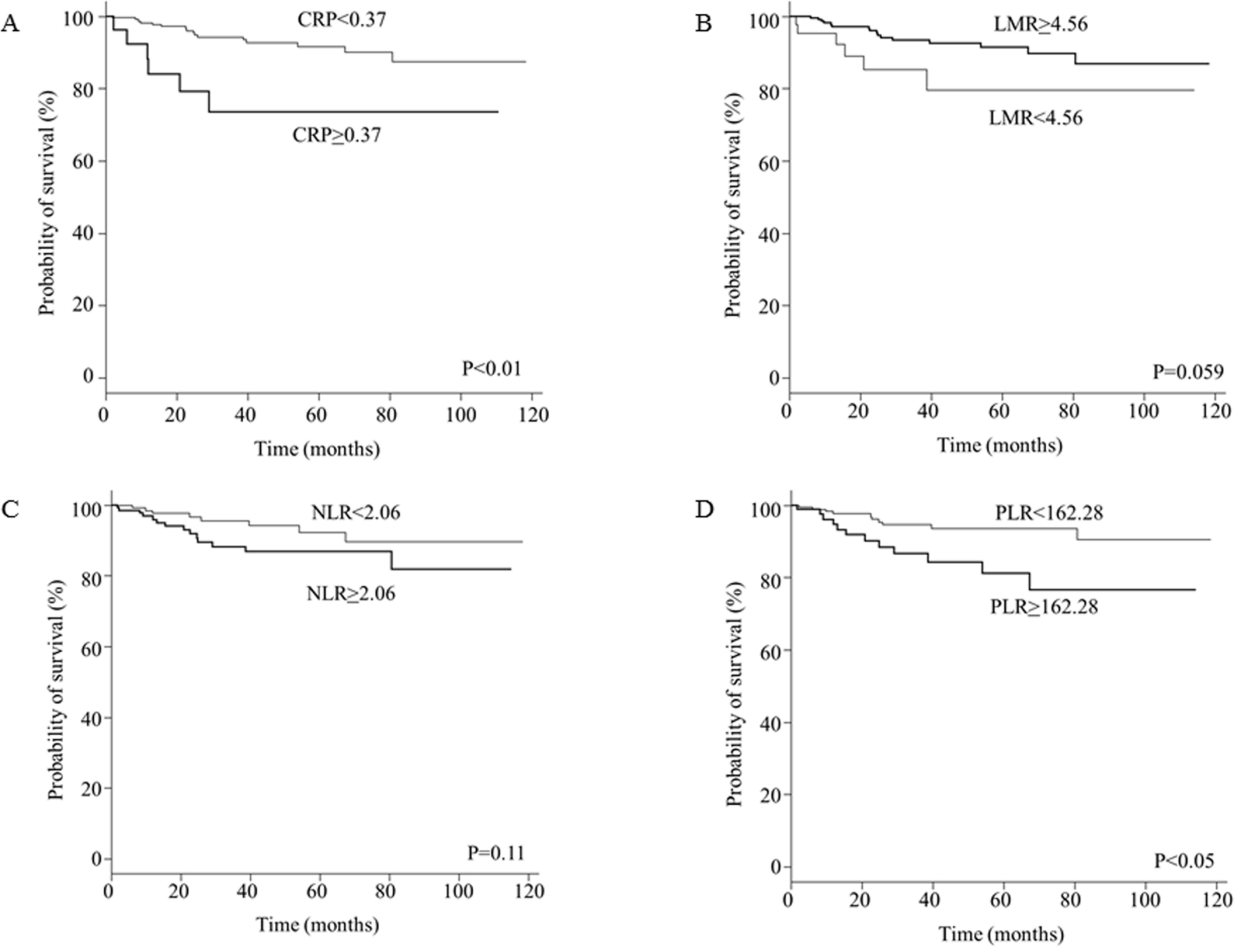
Kaplan-Meier analysis of disease-free survival stratified by inflammation-based parameters in patients with breast cancer. (A) C-reactive protein (CRP); (B) neutrophil-to-lymphocyte ratio (NLR); (C) lymphocyte-to-monocyte ratio (LMR); and (D) platelet-to-lympho cyte ratio (PLR).

The mean follow-up period was 41 months, during which 22 patients (7%) experienced recurrence. The DFS curves according to CRP, LMR, NLR, and PLR are shown in Fig 1A–1D. The DFS rate was significantly lower in the CRP-high group than in the CRP-low group (5-year DFS: 73.6% vs. 91.6%, respectively; P<0.01) as well as in the PLR-high group than in the PLR-low group (5-year DFS: 81.1% vs. 93.6%, respectively; P<0.05). There was no significant difference between the NLR-high and NLR-low groups (5-year DFS: 86.7% vs. 92.3%, respectively; P = 0.11), or between the LMR-high and LMR-low groups (5-year DFS: 91.6% vs. 79.6%, respectively; P = 0.059) (Fig 1).

Univariate analysis revealed a significant impact of the tumor size, lymph node involvement, nuclear grade, CRP, and PLR on DFS. On multivariate analysis, the nuclear grade, CRP, and PLR were independently correlated with poor prognosis (Table 4).

**Table 4:** Survival analyses of clinicopathological factors and inflammation-based scores.

Variables	Univariate analysis		Multivariate analysis	
	Hazard ratio (95% CI)	P-Value	Hazard ratio (95% CI)	P-Value
Age (years)		0.36		
<50	1			
≥50	0.65 (0.25–1.66)			
Tumor size				
<20 mm	1	0.01	1	0.17
≥20 mm	3.72 (1.37–10.14)		2.14 (0.72–6.36)	
ER				
Negative	1	0.057		
Positive	0.4 (0.16–1.02)			
PgR				
Negative	1	0.09		
Positive	0.48 (0.19–1.14)			
HER2				
Negative	1	0.27		
Positive	1.75 (0.64–4.75)			
LN				
Negative	1	0.015	1	0.06
Positive	2.84 (1.22–6.59)		2.32 (0.97–5.58)	
Nuclear grade				
1–2	1	0.01	1	0.01
3	2.92 (1.26–6.79)		3.07 (1.26–7.49)	
CRP (mg/dL)				
<0.37	1	0.004	1	0.04
≥0.37	3.97 (1.55–10.17)		2.85 (1.03–7.89)	
LMR				
<4.56	1	0.06		
≥4.56	0.4 (0.16–1.04)			
NLR				
<2.06	1	0.11		
≥2.06	2.03 (0.85–4.84)			
PLR				
<162.28	1	0.01	1	0.035
≥162.28	2.94 (1.27–6.82)		2.61 (1.07–6.36)	

Abbreviations: CI: confidence interval; CRP, C-reactive protein; LMR, lymphocyte-to-monocyte ratio; NLR, neutrophil-to-lymphocyte ratio; PLR, platelet-to-lymphocyte ratio; ER, estrogen receptor; PgR, progesterone receptor; HER2, human epidermal growth factor receptor 2; LN, lymph node involvement.

## Discussion

Since tumor progression involves its interaction with inflammatory response molecules in its microenvironment [12], many inflammatory molecule-based scoring systems have been evaluated as prognostic indicators in various malignant tumors [5–8]. To the best of our knowledge, ours is the first study to show that elevated preoperative CRP and PLR values are the most reliable prognostic indicators in patients with breast cancer among such inflammatory response markers.

Neutrophils suppress the cytolytic activity of lymphocytes, natural killer cells, and activated T-cells [13]. Furthermore, tumor-associated neutrophils promote remodeling of the extracellular matrix, which results in the release of basic fibroblast growth factor, the migration of endothelial cells, and the dissociation of tumor cells from their primary mass. These events ultimately result in enhanced angiogenesis, tumor growth, and progression to a metastatic phenotype [14, 15].

Monocytes can differentiate into tumor-associated macrophages in the tumor microenvironment. Tumor-associated macrophages are actively recruited at the tumor site by tumor-derived chemotactic factors, where they accelerate tumor progression through the production of growth factors and cytokines that lead to angiogenesis and anti-immune responses [7, 16].

Platelets can accumulate following the stimulation of megakaryocytes by inflammatory mediators that are released by the tumor or its microenvironment, such as interleukin (IL)-1, IL-3 and IL-6 [17]. Such platelets can express elevated levels of platelet-derived growth factor, vascular endothelial growth factor, and platelet factor 4, which can stimulate tumor cell proliferation and adhesion to other cells and lead to tumor growth and metastasis [18].

Lymphocytes have a major role in cancer immune-surveillance that targets tumor cell proliferation and metastasis [19]. With the help of CD4^+^ T-cells, CD8^+^ T-cells can control tumor growth by their cytotoxic activity and the induction of apoptosis in tumor cells [20].

Taken together, elevated neutrophils, monocytes, and platelets, as well as lower lymphocyte levels, may correspond to tumor aggressiveness, and constitute adverse prognostic biomarkers in various cancers. Therefore, it is biologically feasible that a combined index of the NLR, LMR, and PLR could be a novel indicator of malignant potential and prognosis in various tumors.

CRP is a nonspecific inflammatory acute-phase protein, the production of which is upregulated in hepatocytes through exposure to inflammatory cytokines such as IL-1, IL-6, and tumor necrosis factor [21]. These inflammatory cytokines are also known to influence the growth, proliferation, and differentiation of tumor cells [12]. Consistent with previous studies of colorectal [21], esophageal [22], and hepatic carcinomas [8], an elevated serum CRP concentration is an indicator of unfavorable prognosis in patients with breast carcinoma.

Azab et al. [23] reported that the NLR was a predictor of mortality in breast cancer; this was inconsistent with our results. However, their studies involved patients of different geographic regions and races, as well as different cut-off values and survival endpoints. Because Caucasians have higher peripheral blood neutrophil counts than do Asians, as well as lower lymphocyte counts [24], the traditional NLR cut-off value may not be applicable. We have shown that an NLR of 2.06 is the optimal threshold to predict survival by using ROC analysis. Moreover, we used DFS as the endpoint to assess the prognostic value of the NLR in our study; this ought to exclude the influence of non-cancer related deaths on the data.

Several limitations of this study should be acknowledged. First, the study involved a relatively short follow-up period that may have selected only for the fastest-growing tumors, in addition to a small sample size and single-center design. Second, the study’s retrospective design renders it susceptible to selection and analytical biases. Third, the study could have benefitted from a larger sample size. Nevertheless, our data indicated that increased preoperative CRP and PLR might represent an independent prognostic factor in patients with breast cancer.

## Conclusions

Ours is the first study to evaluate the prognostic value of easily obtainable peripheral inflammation-based parameters simultaneously. Our results suggest that the preoperative CRP and PLR values significantly affect DFS in patients with breast carcinoma, and are superior to the NLR and LMR in terms of prognostic reliability. However, further validation and feasibility studies are required to better understand the roles of the preoperative CRP levels and PLR values.

## Supporting information

S1 File Takeuchi. (XLSX)
